# Bacteria-Based Analysis of HIV-1 Vpu Channel Activity

**DOI:** 10.1371/journal.pone.0105387

**Published:** 2014-10-01

**Authors:** Robert Taube, Raphael Alhadeff, Dror Assa, Miriam Krugliak, Isaiah T. Arkin

**Affiliations:** 1 Department of Biological Chemistry, The Alexander Silberman Institute of Life Sciences, The Hebrew University of Jerusalem, Edmund J. Safra Campus, Jerusalem, Israel; 2 Institue of Biology, Humboldt-Universität zu Berlin, Berlin, Germany; Helmholtz Zentrum Muenchen - German Research Center for Environmental Health, Germany

## Abstract

HIV-1 Vpu is a small, single-span membrane protein with two attributed functions that increase the virus' pathogenicity: degradation of CD4 and inactivation of BST-2. Vpu has also been shown to posses ion channel activity, yet no correlation has been found between this attribute and Vpu's role in viral release. In order to gain further insight into the channel activity of Vpu we devised two bacteria-based assays that can examine this function in detail. In the first assay Vpu was over-expressed, such that it was deleterious to bacterial growth due to membrane permeabilization. In the second and more sensitive assay, the channel was expressed at low levels in K^+^ transport deficient bacteria. Consequently, Vpu expression enabled the bacteria to grow at otherwise non permissive low K^+^ concentrations. Hence, Vpu had the opposite impact on bacterial growth in the two assays: detrimental in the former and beneficial in the latter. Furthermore, we show that channel blockers also behave reciprocally in the two assays, promoting growth in the first assay and hindering it in the second assay. Taken together, we investigated Vpu's channel activity in a rapid and quantitative approach that is amenable to high-throughput screening, in search of novel blockers.

## Introduction

Vpu is a small accessory protein found in HIV-1 [1] that was shown to increase the transmission efficiency of the virus [2]. While containing only 81 amino acids, Vpu increases viral release via two distinct mechanisms: (i) Down regulation and degradation of CD4, the receptor that mediates viral endocytosis; and (ii) Functional inactivation of BST-2, part of interferon-dependent antiviral response pathway.

CD4 is the receptor that is recognized by the viral spike glycoprotein GP160 [3], [4]. However, during the synthesis of new viral progeny, CD4 interacts with GP160 in the ER to form trafficking-incapable complexes that hinder viral maturation [5]–[7]. Vpu inhibits this process by causing rapid degradation of CD4 [8], [9], thereby facilitating viral protein maturation.

BST-2 (or tetherin) is part of the cellular anti-viral defense system that theaters mature viral progeny to lipid rafts. It inhibits not only HIV-1 but also other viruses such as Lassa and Marburg virus [10]. In HIV-1 BST-2's activity is neutralized by Vpu [11], [12] in a mechanism that is not entirely understood. Vpu is also known to impact cellular apoptosis due to its ability to deregulate I*κ*B [13]. Consequently, it induces apoptosis due to the negative impact it has on NF*κ*B [14]. However, our current understanding places Vpu's contribution to HIV-1 infectivity, mostly on its antagonistic role versus BST-2.

The small size of Vpu and its oligomeric nature prompted suggestions that it might be an ion channel [15]. Experiments later on have shown that the protein does indeed possess cation channel activity [16], for which the transmembrane domain of the protein is responsible for [17]–[20]. Finally, the channel activity of the protein could be inhibited by 5-(N,N-hexamethylene)amiloride (HMA) [21].

Despite the aforementioned evidence, it is not at all clear if Vpu's channel activity has any impact on its ability to enhance virus release. For example, some mutations in Vpu, and known blockers that impair channel activity do not affect viral release [22], [23]. It is worth noting that there are reports from commercial entities of a blocker that does posses anti-viral activity [24]. Furthermore, mutations that do impact viral release retain native channel activity [25]. However, when the transmembrane domain of Vpu was replaced with that of another viroporin, the M2 H^+^ channel from Influenza, the resulting virus was inhibited by rimantadine (a known M2 blocker) [26].

In order to gain further insight into the role of Vpu's channel activity it is important to be able to assay this functionally quantitatively and rapidly. Membrane conductivity may be measured by numerous techniques, some of which provide the most detailed data on any macromolecular system [27]. For example, both black lipid membranes and patch clamping offer outstanding sensitivity and the ability to record the behavior of single channels. At times conductivity may even be simulated computationally [28], [29]. However, both techniques are not readily amenable to screening a large number of compounds due to their inherent complexity. Electrophysiological data may also be recorded by other means, such as solid-supported membrane technology [30], as well as indirect approaches that rely on ion sensitive fluorescence markers [31].

Another approach to measure channel activity is a cell based assay, in which the channel is heterologously expressed in a host cell. If the growth of the host cell is impacted, it may be used as direct indicator of channel activity.

Kurtz and co-workers have constructed such a system in *Saccharomyces cerevisiae*, whereby over-expression of Influenza M2 H^+^ channel resulted in growth inhibition [32]. Another study in *Saccharomyces cerevisiae* was conducted by González and coworkers, showing K^+^ transport activity of Vpu [33]. We established a similar system in *Escherichia coli*, and the ease in which bacteria can be manipulated enabled us to use the assay quantitatively [34]. More recently, a very similar system was introduced by Inouye and co-workers, that differed principally in the choice of the chimeric construct [35].

All of the above assays, are constructed in such a way that the channel activity of the protein is harmful to the host. Herein, we develop an entirely new assay in which the channel activity is beneficial to the bacteria and is thereby more sensitive. Together, we employ both assays towards the characterization of Vpu and examine its interaction with its cognate blockers.

## Results

In order to investigate the channel activity of Vpu we made use of two bacteria-based assays. In the first assay, the channel negatively impacts bacterial growth, while in the second assay it is vital to the bacteria. Together, the two assays enable a simple and accurate analysis of the channel activity of Vpu and it cognate blockers. All protein expression is done when the channel is fused to the carboxy-terminus of the Maltose binding protein (MBP), thereby forming a chimera.

### Negative bacteria-based assay

The first bacteria-based assay that we used was previously tested by us on the Influenza M2 H^+^ channel [34], [36], [37]. In this assay the protein is expressed in bacteria at relatively high levels, resulting in growth retardation due to membrane permeabilization.

**Figure 1:**
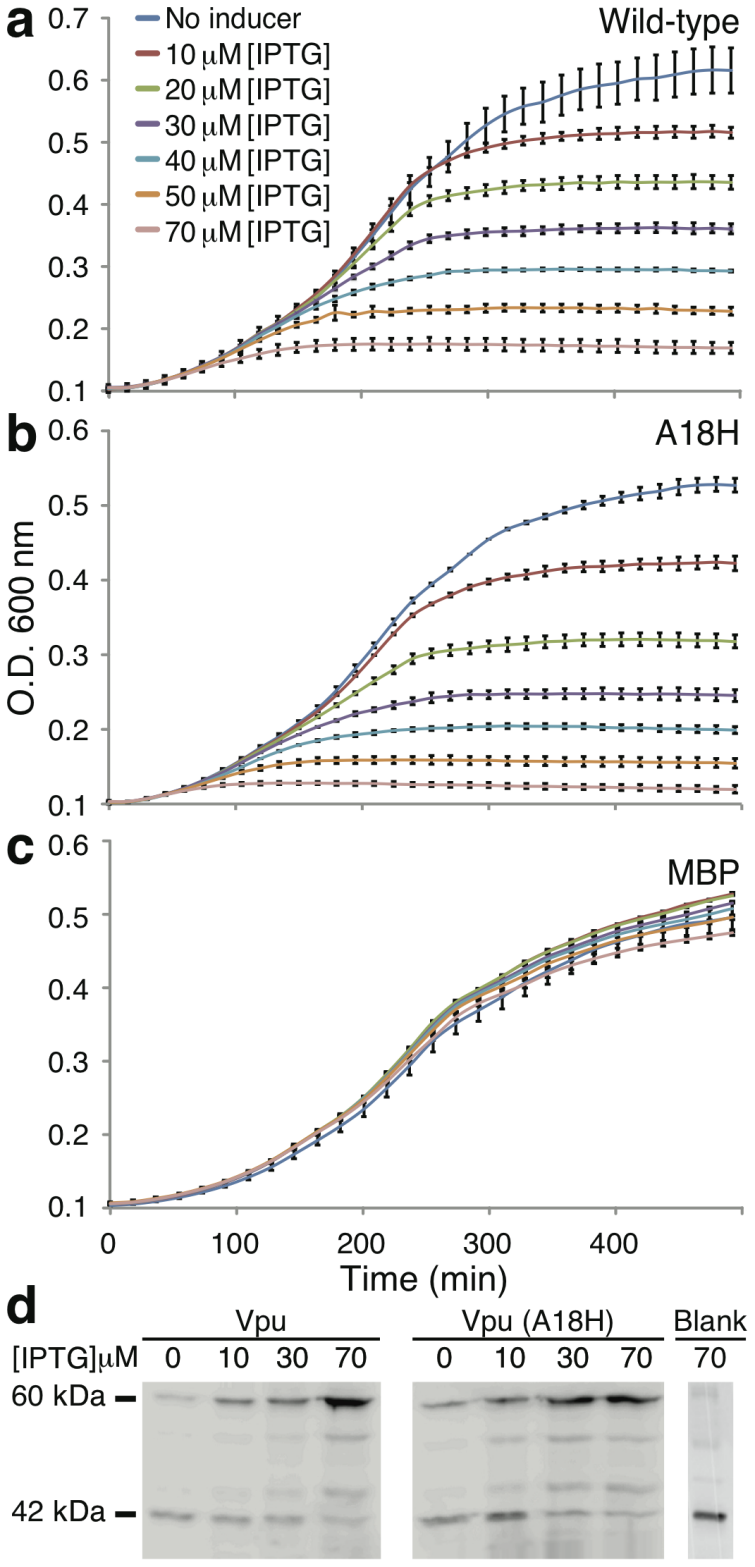
Growth curves of DH10B bacteria expressing two different Vpu channels: wild-type (a) and the A18H mutant (b) as a function of inducer concentration (IPTG). Both channels are expressed as a chimeric construct fused to the C-terminus of the maltose binding protein (MBP). Panel c, as a control, depicts the same experiment but with MBP without a channel attached to it. Panel d presets a Western blot analysis (using anti-MBP antibody) of chimeric protein expression at different concentrations of the IPTG inducer. An empty plasmid, as a negative control is presented to show expression of native MBP protein in the bacteria (labeled blank).

In Fig. 1a we show the impact of Vpu expression on *Escherichia coli* growth. Increased concentration of the inducer Isopropyl-*β*-D-thiogalactopyranoside (IPTG), which in turn results in higher Vpu expression, causes significant growth impairment. A similar scenario is found with the A18H mutant of Vpu (Fig. 1b). This mutant was identified as one that can be blocked by rimantadine [38], a feature that will be used by us below. Note that expression of the MBP alone, is not detrimental to the bacteria, as shown in Fig. 1c. Finally, direct visualization of protein expression levels using Western blotting, mirrors the above results (Fig. 1d).

**Figure 2:**
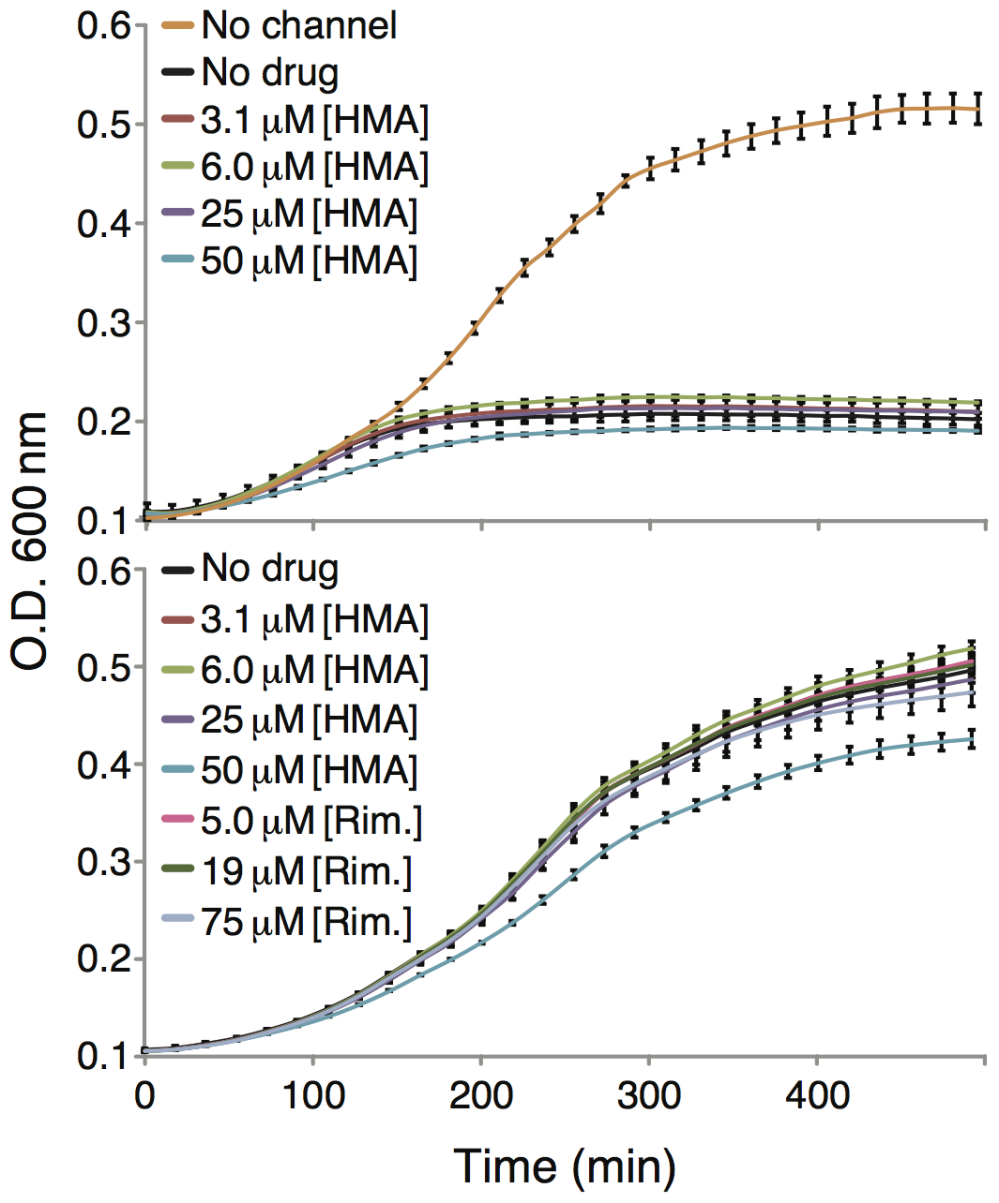
Top. Growth curves of DH10B bacteria expressing MBP-Vpu chimera as a function of different HMA concentrations. The bottom panel depicts a toxicity analysis of HMA and rimantadine (Rim.) on bacteria that express MBP without a channel attached to it.

In order to show that the growth impairment results from the channel activity of the protein we made use of HMA, a known blocker of Vpu [21]. Yet, results shown in Fig. 2 indicate that HMA has little, if any impact on bacterial growth. In other words, HMA is not able to alleviate the negative impact growth of Vpu.

**Figure 3:**
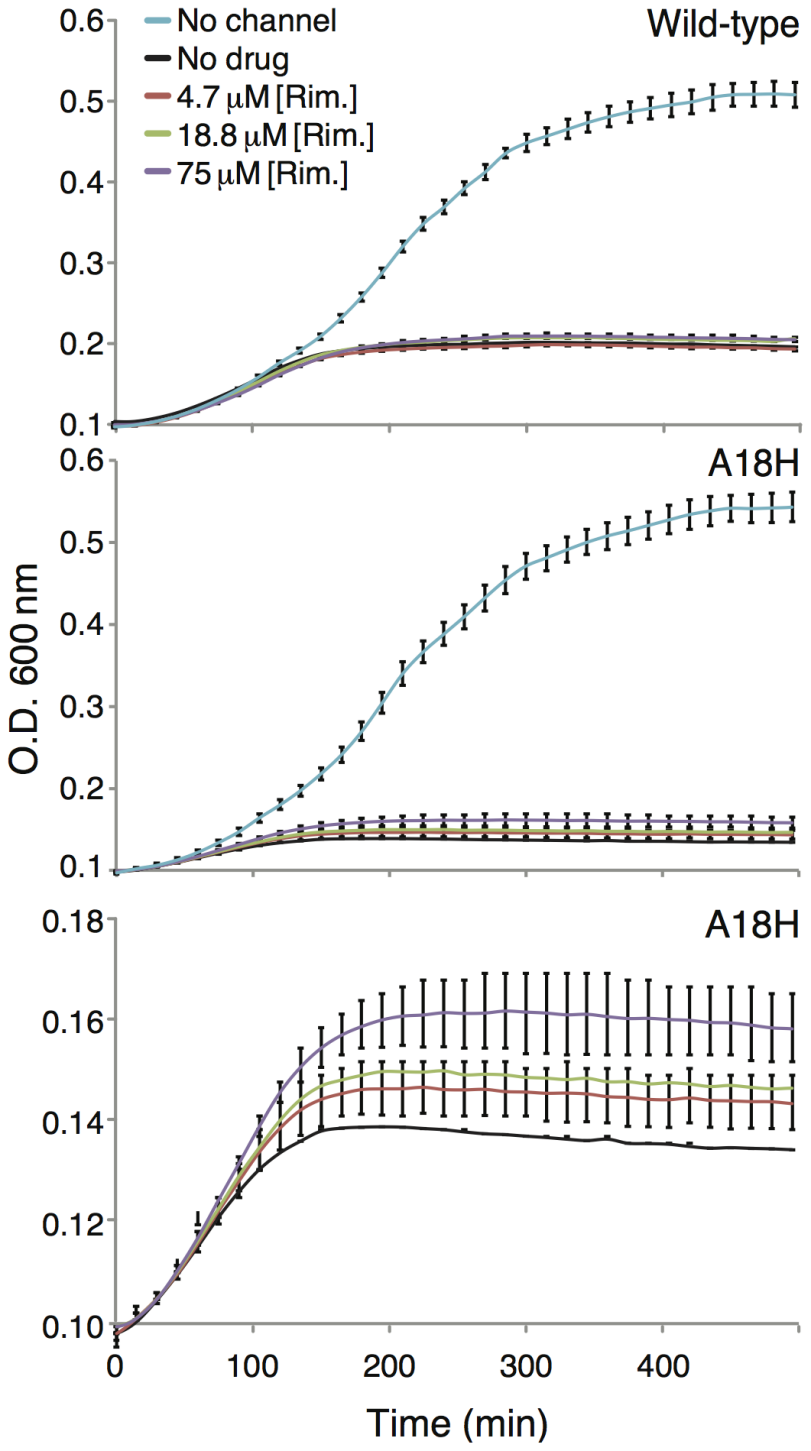
Growth curves of DH10B bacteria expressing wild-type Vpu (top) or the A18H mutant (middle and bottom) as a function of different rimantadine concentrations. The bottom panel is an expansion of the middle panel.

**Figure 4:**
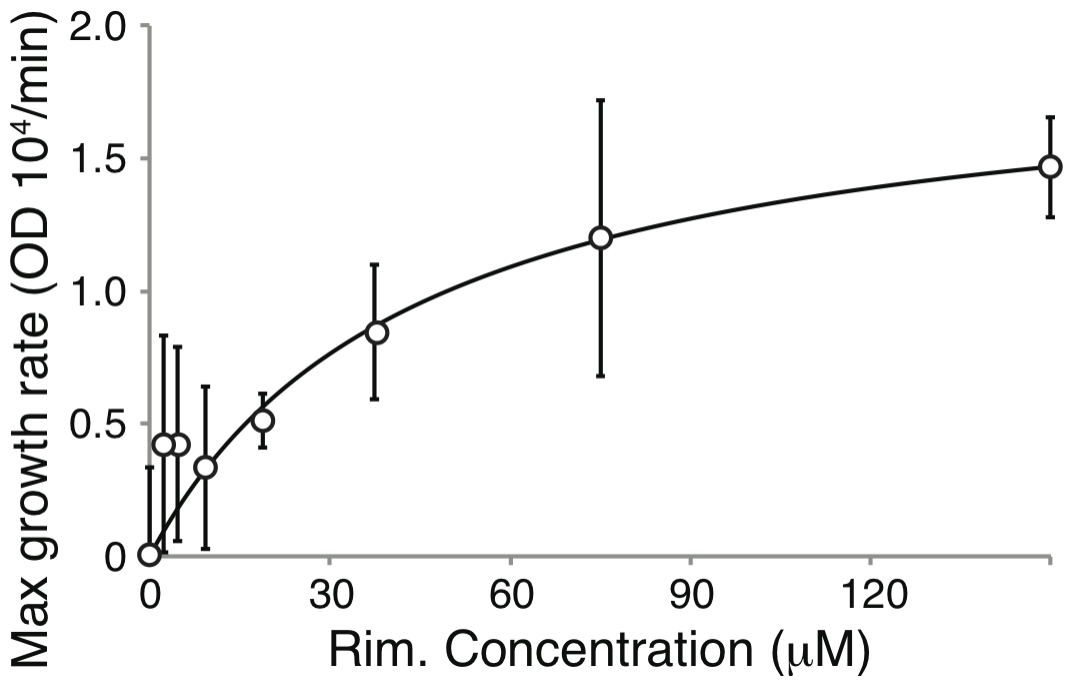
Maximal growth rate of DH10B bacteria expressing the Vpu A18H mutant as a function of different rimantadine concentrations. Fitting according to the Monod equation (solid line), to the experimental data (circles).

Better results were obtained when using the A18H mutant of Vpu that is known to be blocked by rimantadine [38]. In this instance, rimantadine impacted the growth of bacteria that expressed the A18H mutant of Vpu (Fig. 3 middle and bottom panels). Specifically, increased concentrations of rimantadine resulted in diminished growth retardation of the bacteria. The results could then be plotted as a dose response curve (shown in Fig. 4) and when fit to the Monod equation yielded a *K_s_* value of 45 µM. In contrast, no effect of rimantadine was shown on bacteria that express the wild-type Vpu channel (Fig. 3 top panel).

### Positive bacteria-based assay

In order to complement the above assay and increase our sensitivity in detecting channel activity, we decided to design a complementary bacteria-based assay.

**Figure 5:**
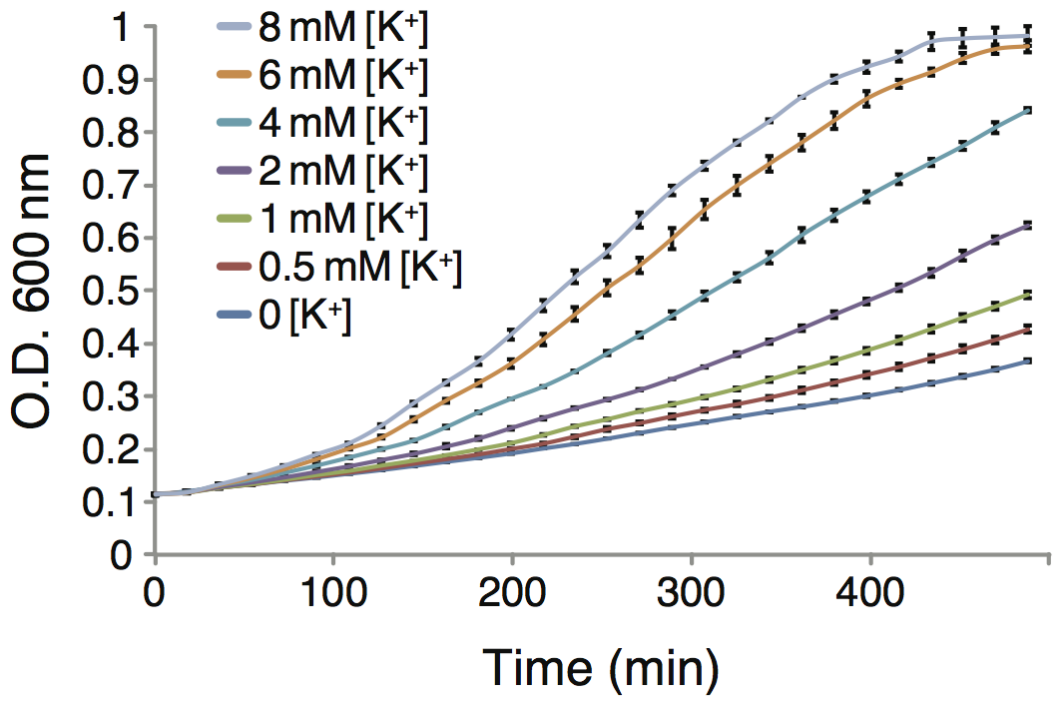
Growth curves of LB650 bacteria as a function of different K^+^ concentration, as noted in the inset. The bacteria are expressing a control plasmid that contains just the MBP.

Towards that end we made use of a special bacterial strain, LB650, that has two K^+^ transport gene knocked out: *trkH* and *trkG* [39]. As shown in Fig. 5, these bacteria grow very poorly in regular LB media, unless the media is supplemented with additional K^+^. For example, values of 8 mM [K^+^] (in additional to basel K^+^ in LB media) restore growth to levels of normal, K^+^ transport competent bacteria.

**Figure 6:**
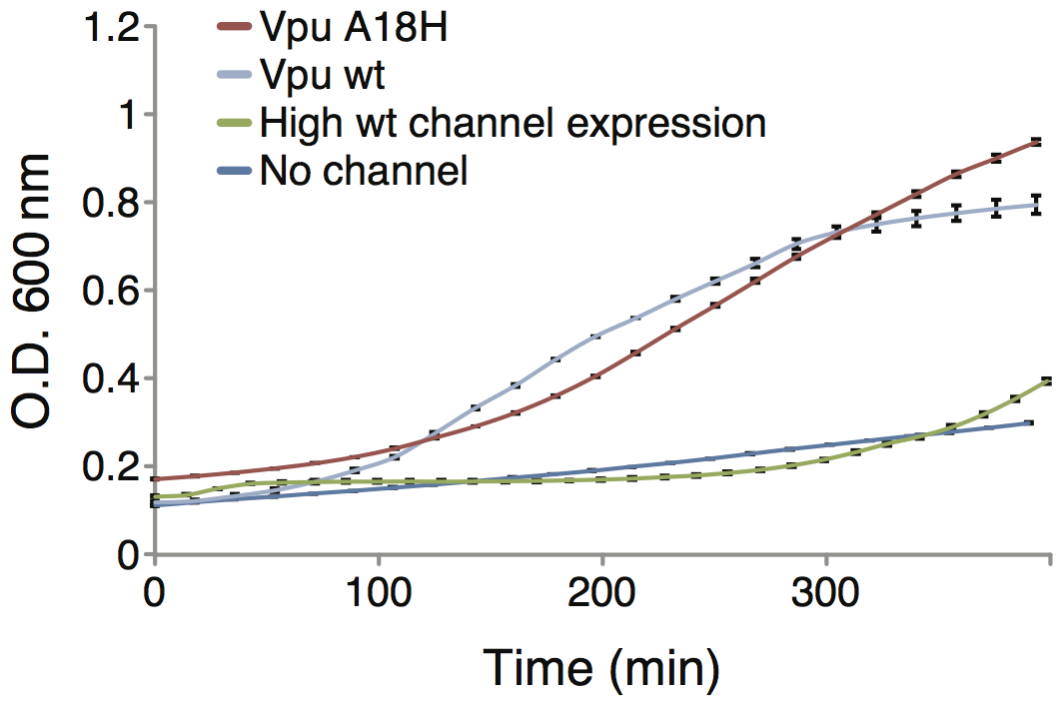
Growth curves of LB650 bacteria expressing two different Vpu channels: wilt-type and the A18H mutant. The wild-type channel was also over-expressed using 50 µM [IPTG]. Bacteria that express the MBP without any channel are used as control. No K^+^ was added to the growth media.

The impact of channel expression in these bacteria can clearly be seen in Fig. 6. Expression of Vpu in the LB650 bacteria enables growth that is similar to that which is obtained with the addition of K^+^. Similar results were obtained when the A18H mutant of Vpu is expressed. Hence, expression of a channel that is able to transport K^+^ enables the K^+^ transport deficient bacteria to grow in otherwise lethal K^+^ concentrations. We note that when Vpu is expressed at high levels, the positive impact on growth is lost and once more the protein is toxic to bacteria (green curve in Fig. 6). This result is reminiscent of the first bacteria-based assay (Fig. 1). One possible explanation might be the collapse of the bacterial proton motive force due to the establishment of a new alternative route for protons to reenter the cytoplasm. Blocking this route will therefore alleviate growth, as seen in Fig. 3.

**Figure 7:**
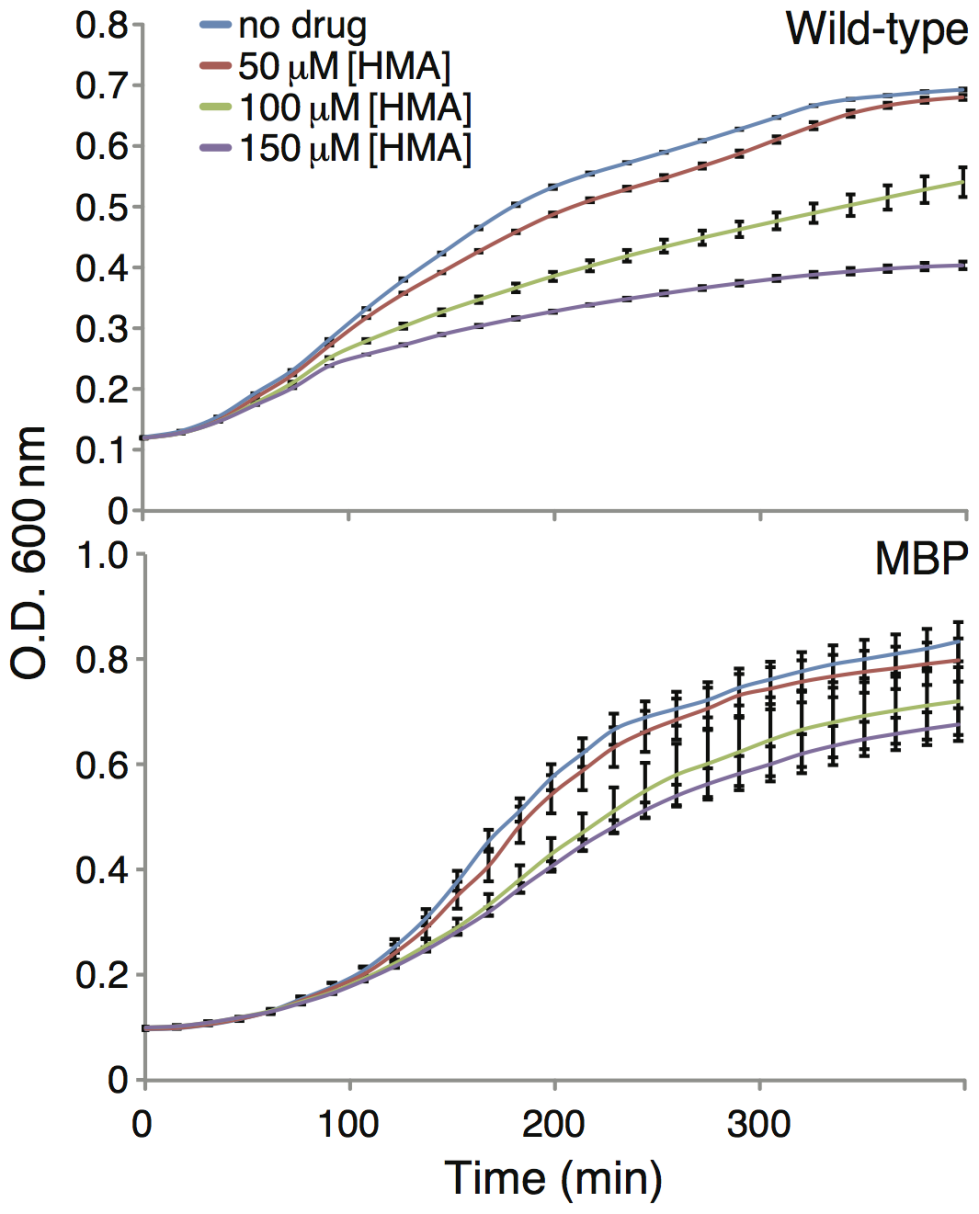
Top. Growth curves of LB650 bacteria expressing wt Vpu as a function of different HMA concentrations. No K^+^ was added to the growth media. Bottom. Growth curves of LB650 bacteria expressing MBP without a channel fused to it (as a control), as a function of different HMA concentrations. K^+^ was added to the growth media to final concentration of 87 mM.

In the current assay the channel exerts a positive impact on bacterial growth. Hence we would expect that any blocker would be detrimental to bacterial viability. Our results are consistent with this hypothesis. In Fig. 7 we show that HMA, a known blocker of Vpu [21], negates the positive effect that Vpu has on bacterial growth. We note that while HMA is somewhat toxic to bacteria (see control in the bottom panel of Fig. 7), its negative impact on Vpu expressing, LB650 bacteria goes beyond that.

**Figure 8:**
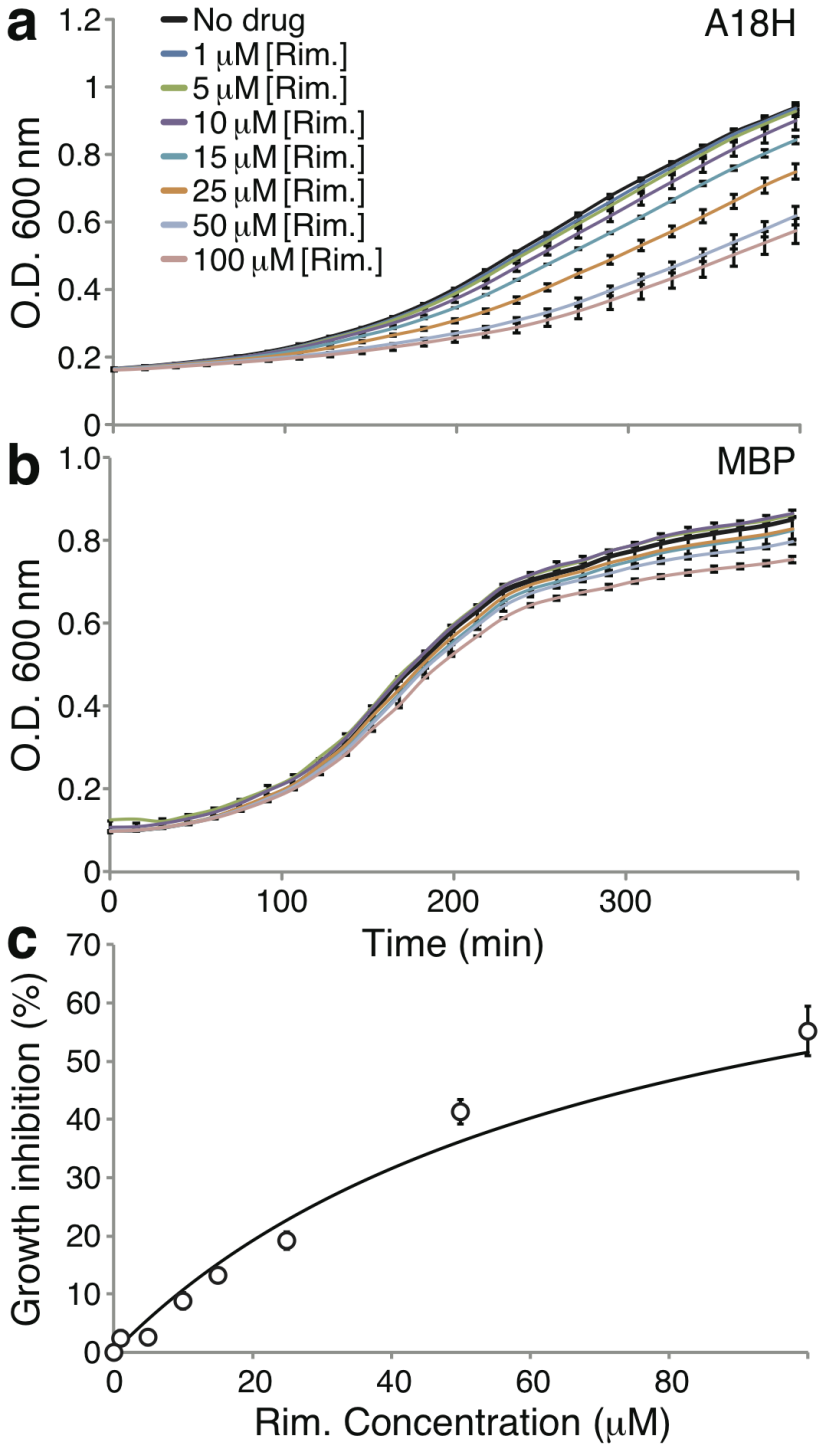
Impact of rimantadine on bacterial growth. a. Growth curves of LB650 bacteria expressing mutant H18A Vpu as a function of different rimantadine concentrations. No K^+^ was added to the growth media. b. Growth curves of LB650 bacteria expressing MBP without a channel fused to it (as a control), as a function of different rimantadine concentrations. K^+^ was added to the growth media to final concentration of 87 mM. c. Fitting of the data in panel a according to the Monod equation (solid line), to the experimental data shown above (circles).

A similar analysis is shown in Fig. 8a. In this instance the positive impact upon growth of a channel that contains the A18H mutation is negated with rimantadine. In this instance, the majority of the negative activity of rimantadine may be attributed to its impact on the channel since it exhibits minimal toxicity (Fig. 8b). This particular mutation was previously shown to render viruses that harbor the mutated Vpu channel activity sensitive to rimantadine [38]. Finally the growth inhibition data of rimantadine on bacteria that express the Vpu A18H mutant could be fit according to the Monod equation to obtain a *K_s_* value of 73 µM (Fig. 8c). We note that no impact on growth of bacteria that express the wild-type Vpu channel is obtained by rimantadine (data not shown).

## Discussion

The goal of this study was to investigate the ion channel activity of Vpu. The assay used must be both accurate to be used in detailed analyses, and simple such that it can be employed in high-throughput screening. Based on these guiding considerations we constructed two bacteria-based assays and used them to examine wild-type Vpu, a known mutant and the effects of channel blockers thereupon.

It is worth pointing out the key differences between the two assays. In both instances a channel is expressed in bacteria, yet an opposite phenotype is sought. In the negative assay, channel expression leads to bacterial death, while in the positive assay it leads to growth. The result of any channel blocker in the two assays will also have an opposite effect. In the negative assay, when the channel is detrimental to the bacteria, the activity of channel blocker will enable growth. In contrast, in the second assay when the channel is beneficial to the bacteria, blockers will be detrimental to growth. Hence, the first assay can be viewed as a genetic screen for channel blockers while the second assay is a genetic screen that can identify channels.

The key mechanistic differences that enable the aforementioned opposite phenotype are as follows: (i) In the negative assay the channel is expressed at high levels, which results in membrane permeabilization. In contrast, in the second assay the channel is expressed at levels which are sufficiently low, so as not to hamper the proton motive force. (ii) In the second assay the channel is expressed in a K^+^ transport deficient bacteria. In this way the heterologously expressed channel provides a benefit. The first assay makes use of standard *Escherichia coli*.

The results that we obtained are consistent with the assay construction. In the negative assay Vpu was expressed at various levels in *Escherichia coli*. As we have shown previously with the M2 channel from Influenza [34], [36], [37], increased expression of a channel in bacteria results in substantial growth retardation. The extent of growth inhibition can easily be controlled by the amount of inducer (Fig. 1). The cause of growth impairment is most likely a collapse of the proton motive force that is at the heart of bacterial bioenergetics [40]. The reader is referred to the following reviews for a comprehensive discussion on viroporins and their effects on membrane permeabilization [41], [42].

In order to complement the above assay and increase our sensitivity in detecting channel activity, we decided to design a different bacteria-based assay. In the previous assay, channel activity resulted in impaired bacterial growth. In the second assay we sought an opposite setting, whereby channel activity would result in enhanced growth.

The positive bacteria based assay is based on the LB650 K^+^ transport deficient bacteria strain [39]. These bacteria are incapable of growing at very low K^+^ concentrations (Fig. 5). However, when we express Vpu in these bacteria we show that appreciable growth can be obtained (Fig. 6).

In both assays it is important to show that the impact upon bacterial growth results from the channel activity of the expressed protein. Specifically, in the negative assay, protein expression may be harmful to bacteria due to numerous reasons. In the past, using the same assay, we have shown that the deleterious effect on growth imposed by the influenza M2 H^+^ channel could be partially alleviated by its cognate amino-adamantyl blockers [34], [36], [37]. Therefore, in the current study we tried to make use of HMA [21], a known blocker of Vpu. However, no effect by HMA was found (Fig. 2).

It is possible to speculate why HMA was not able to revert the harmful impact of Vpu on bacterial growth: (i) HMA binding and inhibition of Vpu may not be sufficient. For example, rimantadine was only able to alleviate about 50% of the negative growth impact of the influenza M2 channel, despite being a nano-molar blocker [34], [36], [37]. (ii) HMA might not be able to successfully block the Vpu channel due to limited access in the bacteria-based assay. (iii) Alternatively, it is still possible that the negative impact upon bacterial growth of Vpu has little to do with its channel activity an therefore HMA does not affect it.

The latter possibility was shown to be false using a special mutant of Vpu, A18H that is known to be sensitive to rimantadine [38]. In this instance the blocker was able to alleviate, albeit to a minor extent, the negative impact upon growth of the channel (Fig. 3). The results were sufficiently quantitative and were fit to a Monod dose response curve, yielding a *K_s_* value of 73 µM (Fig. 4)

The impact of the channel blockers was more successfully detected in the positive assay. In this approach, an effective blocker eliminates the positive impact upon growth of the channel. For example, HMA was shown to negatively impact bacterial growth in a dose dependent manner in the LB650 bacteria (Fig. 7). This results is in contrast to the lack of effect of HMA in the positive assay (Fig. 2).

It is possible to speculate that the positive assay is more sensitive in detecting the effect of channel blockers using the following rationale: It is easier to eliminate a positive influence on growth as opposed to block a detrimental factor. Thus, relatively poor channel blockers will only be detected in the positive assay.

When the blocker is relatively efficacious, it is detected by both assays. Such is the case with rimantadine that blocks the A18H Vpu mutant [38]. In the negative assay it revived bacterial growth (Fig. 3), while in the positive assay it resulted in slower growth (Fig. 8). Furthemore, in both instances the *K_s_* values of the blocker were relatively similar: 45 µM versus 73 µM in the negative (Fig. 4) and positive (Fig. 8) assays, respectively. However, it is clear that the sensitivity of the positive assay is much larger than that of the negative assay.

In conclusion, we show using two different approaches that Vpu exhibits channel activity. The channel activity of the wild-type protein can be blocked by HMA and that of the A18H mutant by rimantadine. The assays used are readily amenable to high-throughput screening and provide a potential route to identify new Vpu blockers.

## Materials and Methods

### Chemicals

Isopropyl-*β*-D-thiogalactopyranoside (IPTG) was purchased from Biochemika-Fluka (Buchs, Switzerland). 5-(N,N-Hexamethylene)amiloride, Rimantadine and all other chemicals were purchased from Sigma-Aldrich (Rehovot, Israel).

### Plasmids and bacterial strains

The Vpu gene (according to the sequence in [1]) was cloned in the pMal-p2x commercial plasmid (New England BioLabs, Ipswich, MA) that carries a p-lac promotor and antibiotic resistance. The plasmid is designed to over express a fusion-taged maltose binding protein (MBP) on the N-terminus of the protein of interest, which in the current study is a codon optimized gene coding for Vpu of HIV-1 [43]. The chimera further carries a His-Tag at the C-terminus. This chimera construct was used throughout the study.

Two main strains of *Escherichia coli* K12 were used: DH10B and LB650. DH10B bacteria cells were purchased from Invitrogen (distributed by Rhenium, Modi'in Israel). LB650 bacteria were a kind gift of Prof. K. Jung (Ludwig-Maximilians Universität München) and Prof. G.A. Berkowitz (University of Connecticut). The bacteria carry deletions in genes connected to potassium uptake [39].

### Bacteria growth

To investigate the impact of Vpu expression upon bacterial growth rates 25 ml Medium (containing 100 µg/ml ampicillin) were inoculated with 0.25 ml overnight culture and grown until O.D.600 of 0.1. The LB650 bacterial strain was grown with the addition of 10 mM KCl to the medium. When the culture reached O.D.600 0.1 the culture was centrifuged for 8 min with 4000 rpm twice and the pellet was washed twice in regular LB-medium [44] (*i.e.* no supplemented K^+^). IPTG concentration used for induction was 50 µM unless specified otherwise. Media used for induction was identical to the previous growth media except for the addition of IPTG at the appropriate concentration. All inhibitors were solvated in water. Finally, all experiments were conducted in duplicates or triplicates.

The specific growth conditions were monitored in 96-well-microtiter-plates. 5 µl additives of 40-fold of the final concentration were dissolved with 195 µl of bacterial culture. The O.D.600 was measured with the plate reader Infinite F200 pro (Tecan, Switzerland). The program was chosen as shaking orbitally, 250 rpm in 37°C, measured every 17 minutes at 600 nm. Note that the optical path was not equal to 1 cm.

### Western blotting

DH10B bacteria harboring the pMAL-p2X-Vpu wild-type plasmid were incubated overnight, diluted and grown to O.D.600 of 0.07–0.1. Identical analyses were conducted with plasmids that contained the Vpu A18H mutant, and without the MBP gene (as a reference). The bacteria were then transferred to tubes, and IPTG was added to final concentrations as stated. After incubation in 30°C for three hours bacteria were harvested and the amount of bacteria in each sample was equated according to their O.D.600. The pellet was resuspended in 750 uL Lysis buffer (50 mM Tris pH = 8.0, 10?% glycerol, 0.1 % Triton X-100, 1 mM phenylmethylsulfonyl fluoride, 0.2 mg/ml lysozyme and 50 µg/ml DNase I) and then sonicated three times for 30 seconds at 39 W (vibracell by sonics, Newtown, CT). The lysates were centrifuged, and 40 µl were harvested from the supernatant and 10 µl of concentrated sample buffer (×5) were added.

The samples were boiled (95°C, 5 min) and loaded onto a precast Any kD Mini-PROTEAN TGX Gels (Bio-Rad Laboratories, Inc., Hercules, CA) and electrophorated for 35 min under 70 mA. The marker was Precision Plus Protein Dual Color Standards (Bio-Rad). The proteins were transferred onto a nitrocellulose membrane using the Trans-blot Turbo Transfer System (Bio-Rad). Visualization of the MBP chimeras (and endogenic MBP) was possible via blotting with a rabbit antiserum prepared by immunizing against MBP (New England BioLabs Inc., Ipswich, MA) and goat-anti-rabbit, HRP-linked antibody from Abcam (Cambridge, UK).

### Inhibitory constant derivation

The calculation of Monod coefficients (*K_s_*) was obtained by measuring the dose response effect of rimantadine. The resulting data were non-linearly fit according to the Monod equation relating either the growth rate (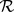) or inhibition to the drug concentration: 
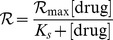



Control data (i.e. data without any drug) were subtracted from the results in order to serve as a reference.
